# Data for proteome analysis of *Bacillus lehensis* G1 in starch-containing medium

**DOI:** 10.1016/j.dib.2017.07.026

**Published:** 2017-07-14

**Authors:** How Lie Ling, Zaidah Rahmat, Abdul Munir Abdul Murad, Nor Muhammad Mahadi, Rosli Md. Illias

**Affiliations:** a Department of Bioprocess and Polymer Engineering, Faculty of Chemical and Energy Engineering, Universiti Teknologi Malaysia, 81310 Skudai, Johor, Malaysia; b Department of Biotechnology and Medical Engineering, Faculty of Biosciences and Medical Engineering, Universiti Teknologi Malaysia, 81310 Skudai, Johor, Malaysia; c School of Biosciences and Biotechnology, Faculty of Science and Technology, Universiti Kebangsaan Malaysia, 43600 Bangi, Selangor, Malaysia; d Malaysia Genome Institute, Ministry of Science, Technology and Innovation Malaysia, Jalan Bangi, 43000 Kajang, Selangor, Malaysia

## Abstract

*Bacillus lehensis* G1 is a cyclodextrin glucanotransferase (CGTase) producer, which can degrade starch into cyclodextrin. Here, we present the proteomics data of *B. lehensis* cultured in starch-containing medium, which is related to the article “Proteome-based identification of signal peptides for improved secretion of recombinant cyclomaltodextrin glucanotransferase in *Escherichia coli*” (Ling et. al, in press). This dataset was generated to better understand the secretion of proteins involved in starch utilization for bacterial sustained growth. A 2-DE proteomic technique was used and the proteins were tryptically digested followed by detection using MALDI-TOF/TOF. Proteins were classified into functional groups using the information available in SubtiList webserver (http://genolist.pasteur.fr/SubtiList/).

**Specifications Table**

**Table:** 

Subject area	Biology
More specific subject area	Microbial proteomics
Type of data	Tables, Figures
How data was acquired	2-DE, MALDI-TOF/TOF (Bruker)
Data format	Raw, Analyzed
Experimental factors	*B. lehensis* grown on starch-containing medium
Experimental features	The extracellular proteins were collected by trichloroacetic acid precipitation of culture supernatant. The protein samples were digested with trypsin and resulting peptides were subjected to MALDI-TOF/TOF and database searching using Mascot.
Data source location	Universiti Teknologi Malaysia, Johor Bahru, Malaysia
Data accessibility	Data is with this article
	Attached supplementary documents

**Value of the data**

• This data set will be of value for the scientific community working in the area of *Bacillus* species since it represents the secreted proteins by *Bacillus* sp. in response to starch.
• This data extends the information available for proteome/secretome changes in *B. lehensis* G1 and can be used as a reference for comparative experiments with different carbon sources.
• Further analysis of the data should allow new insights into mechanisms by which *B. lehensis* proteins are released into the extracellular space.

## 1 Data

**Fig. 1:**
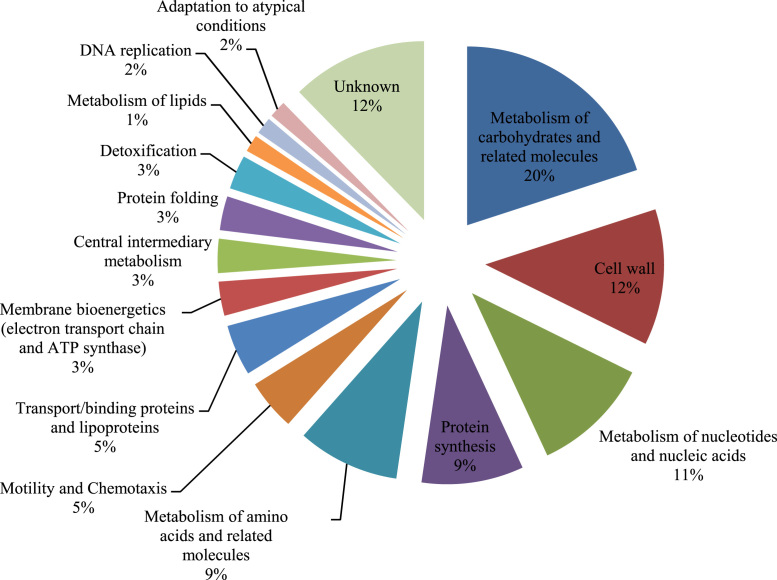
Functional categorization of *B. lehensis* secretome protein identified by MALDI-TOF/TOF.

Extracellular proteins of *B. lehensis* were subjected to 2-DE analysis, producing an extracellular proteome map [1]. A total of 87 identified proteins on the 2-DE was listed in Table 1. Fig. 1 shows the grouping of functional categories of the identified proteins where they are mostly implicated in the metabolism of carbohydrates and related molecules (20%), cell wall (12%), metabolism of nucleotides and nucleic acids (11%) and proteins of unknown function (12%). Supplementary information table shows all assigned peptide sequences detected by MALDI-TOF/TOF analysis for the 87 putative secreted proteins.

**Table 1:** List of the total identified secretome of *Bacillus lehensis* G1 on starch (87 proteins).

Spot no.^a^	Gene no.^b^	Annotation^c^	Theoretical MW (kDa)/pI	Method^d^	Score
1	AIC94431	Hypothetical protein, conserved	72,670.21/4.32	PFF	262
2	AIC95833	Minor extracellular protease	83,878.08/4.1	PFF	221
3	AIC94728	Aconitate hydratase	99,347.96/4.8	PFF	482
6	AIC95721	60 kDa chaperonin	57,311.44/4.73	PFF	57
10	AIC95559	Enolase	46,259.13/4.58	PFF	393
11	AIC95613	Flagella hook-associated protein 1	49,251.02/4.59	PMF	119
12	AIC96376	Hypothetical protein, conserved	38,903.25/4.85	PFF	188
15	AIC93661	Alanine dehydrogenase	39,404.39/5.24	PFF	335
17	AIC93909	Sugar ABC transporter ATP-binding protein	41,000.97/5.38	PFF	97
19	AIC96117	Flagellin	30,592.67/4.52	PFF	219
20	AIC96630	Cysteine synthase	33,028.66/5.24	PFF	254
23	AIC94426	Hypothetical protein, conserved	26,553.07/4.66	PFF	198
24	AIC94046	Chaperone protein DnaK	65,872.55/4.57	PFF	51
26	AIC95608	Flagellar hook-associated protein	66,237.73/5.15	PFF	130
28	AIC96289	Fructose-bisphosphate aldolase	30,779.04/5.04	PFF	56
30	AIC94052	Deoxyribose-phosphate aldolase	23,801.05/5.01	PFF	259
31	AIC94978	Dihydrolipoyllysine-residue acetyltransferase component of pyruvate dehydrogenase complex	46,145.72/4.78	PFF	498
32	AIC95922	GlcNAc-binding protein A	28,951.26/7.25	PFF	80
33	AIC92898	Alkyl hydroperoxide reductase subunit	20,601.02/4.55	PFF	136
35	AIC94804	Ribosome recycling factor	20,882.84/5.81	PFF	60
36	AIC96522	Single-stranded DNA-binding protein	17,548.06/4.98	PMF	92
38	AIC93828	Phage major tail protein	19,786.77/4.63	PFF	117
68	AIC95525	Cysteine desulfurase	44,858/5.25	PFF	91
69	AIC94431	Hypothetical protein, conserved	72,670.21/4.32	PFF	92
70	AIC95782	Sulfatase	74,049.2/4.45	PFF	115
71	AIC93540	Chitinase	62,115.55/4.42	PFF	172
73	AIC96260	ATP synthase subunit alpha	54,711.34/4.89	PFF	78
74	AIC96258	ATP synthase subunit beta	50,931.69/4.89	PFF	172
75	AIC95608	Flagellar hook-associated protein	66,237.73/5.15	PFF	62
76	AIC95608	Flagellar hook-associated protein	66,237.73/5.15	PFF	114
78	AIC94429	Legume lectin, beta chain domain-containing protein	101,452.16/4.55	PFF	67
80	AIC95481	Cytosol aminopeptidase	52,875.15/5.45	PFF	222
81	AIC94131	Fumarate hydratase class II	50,189/5.45	PFF	29
85	AIC96549	Inosine-5'-monophosphate dehydrogenase	51,901.42/5.46	PMF	136
86	AIC96376	Hypothetical protein, conserved	38,903.25/4.85	PFF	70
92	AIC96288	Translaldolase	22,795.16/5.43	PFF	357
96	AIC96376	Hypothetical protein, conserved	38,903.25/4.85	PFF	68
98	AIC95922	GlcNAc-binding protein A	28,951.26/7.25	PFF	92
100	AIC94131	Fumarate hydratase class II	50,189/5.45	PMF	72
101	AIC94216	2-methylcitrate dehydratase	52,815.87/5	PFF	105
102	AIC95918	Trifunctional nucleotide phosphoesterase protein	100,205.5/4.2	PFF	138
103	AIC95608	Flagellar hook-associated protein	66,237.73/5.15	PFF	70
104	AIC95220	Succinate dehydrogenase flavoprotein subunit	64,979.5/5.36	PFF	47
105	AIC96492	Cyclomaltodextrin glucanotransferase	78,624.75/4.72	PFF	339
106	AIC96492	Cyclomaltodextrin glucanotransferase	78,624.75/4.72	PFF	301
107	AIC96492	Cyclomaltodextrin glucanotransferase	78,624.75/4.72	PFF	241
108	AIC93567	Heat shock protein Hsp90	72,131.42/4.74	PFF	279
109	AIC96089	Hypothetical protein, conserved	41,106.33/4.37	PFF	91
110	AIC95790	Xylose isomerase	35,839.84/5.27	PFF	298
111	AIC92706	Endonuclease/CDSuclease/phosphatase	33,963.3/4.37	PFF	176
112	AIC95608	Flagellar hook-associated protein	66,237.73/5.15	PFF	271
115	AIC95591	Hypothetical protein, conserved	39,487.07/5.24	PFF	321
116	AIC96453	Purine nucleoside phosphorylase deoD-type	26,072.75/5.07	PFF	81
117	AIC95591	Hypothetical protein, conserved	39,487.07/5.24	PFF	182
118	AIC94116	Glucokinase	33,294.06/5	PFF	66
120	AIC93828	Phage major tail protein	19,786.77/4.63	PFF	120
121	AIC94609	Nucleoside diphosphate kinase	16,521.72/5.39	PFF	73
122	AIC96118	Hypothetical protein, conserved	21,074.61/4.82	PFF	60
123	AIC96385	Hypothetical protein, conserved	15,073.44/4.32	PMF	79
124	AIC96238	Endopeptidase lytE	52,436.16/5.37	PFF	136
125	AIC96238	Endopeptidase lytE	52,436.16/5.37	PFF	144
126	AIC95662	Zinc D-Ala-D-Ala carboxypeptidase	22,640.86/10.12	PMF	132
127	AIC95945	Cell surface protein	24,644.55/4.83	PFF	46
129	AIC95044	D-alanine aminotransferase	32,232.47/5.45	PFF	218
130	AIC94787	Polyribonucleotide nucleotidyltransferase	78,584.73/4.99	PMF	120
131	AIC93700	Pyridoxal biosynthesis lyase pdxS	31,853.74/5.39	PMF	65
132	AIC96381	Siphovirus tail component	28,326.99/5.2	PFF	53
133	AIC95274	Citrate synthase	41,556.19/5.06	PFF	85
136	AIC95662	Zinc D-Ala-D-Ala carboxypeptidase	22,640.86/10.12	PMF	74
137	AIC94099	Superoxide dismutase [Mn]	22,328.66/5.15	PFF	34
138	AIC96238	Endopeptidase lytE	52,436.16/5.37	PFF	126
139	AIC92651	Elongation factor G	76,489.4/4.88	PFF	41
140	AIC96258	ATP synthase subunit beta	50,931.69/4.89	PFF	80
142	AIC96297	Acetyl-CoA acetyltransferase	41,762.77/5.49	PFF	63
143	AIC95316	Acetyl-CoA synthetase	64,452.18/5.12	PFF	44
144	AIC93282	Endo-beta-1,3-glucanase	31,635.41/4.33	PFF	22
145	AIC94978	Dihydrolipoyllysine-residue acetyltransferase component of pyruvate dehydrogenase complex	46,145.72/4.78	PFF	83
146	AIC94806	Elongation factor Ts	32,345.77/5.06	PFF	69
147	AIC95354	Carbonic anhydrase	20,820.01/5.95	PFF	60
148	AIC93967	Adenine phosphoribosyltransferase	19,084.09/5.16	PMF	100
150	AIC96492	Cyclomaltodextrin glucanotransferase	78,624.75/4.72	PFF	405
151	AIC96492	Cyclomaltodextrin glucanotransferase	78,624.75/4.72	PFF	481
155	AIC92675	Adenylate kinase	24,149.4/4.97	PFF	180
156	AIC95918	Trifunctional nucleotide phosphoesterase protein	100,205.5/4.2	PFF	389
157	AIC95608	Flagellar hook-associated protein	66,237.73/5.15	PFF	160
158	AIC96475	Endo-1,3(4)-beta-glucanase 1	99,760.9/4.53	PFF	248
159	AIC96380	Phage protein	56,563.08/4.6	PFF	398

a Spot number corresponding to spots in Figure S1[1]

b The AIC gene numbering is according to the NCBI taxonomy database for *B. lehensis* G1.

c The annotation was primarily based on the genome annotation of *B. leheniss* G1

d PMF represents the peptide mass fingerprinting using MALDI-TOF MS and PFF represents the peptide fragment fingerprinting using MALDI-TOF/TOF MS

## 2 Experimental design, materials and methods

### 2.1 Preparation of extracellular proteins for proteome analysis

*B. lehensis* G1 extracellular proteins were collected at mid-log phase as previously described [2] with slight modification. Cells were removed from the growth medium via centrifugation at 10,414*g* and 4 °C for 15 min. Proteins in the supernatant were precipitated with 10% (w/v) pre-chilled trichloroacetic acid for 30 min and were collected via centrifugation at 10,414*g* for 15 min. The resulting protein pellet was collected and washed twice with pre-chilled acetone. The supernatant was removed, and the resulting protein pellet was air-dried for 5 min. Finally, the pellet was resolubilized in rehydration buffer (8 M urea, 40 mM dithiotreitol, 2% CHAPS, 0.5% (v/v) carrier ampholytes, 1 mM protease inhibitor cocktail, 0.002% bromophenol blue). The protein concentration of the extracellular protein sample was determined using a 2-D Quant Kit (GE Healthcare, United Kingdom) according to the manufacturer's protocols.

### 2.2 Two-dimensional gel electrophoresis (2-DE), gel analysis, and protein identification

1D isoelectric focusing was carried out using an IEF 100 (Hoefer, United States) and 2D sodium dodecyl sulfate-polyacrylamide gel electrophoresis (Bio-Rad, United States) was conducted using a VS20 WAVE Maxi (Cleaver Scientific Ltd, United Kingdom). The protocols were carried out according to manufacturer recommendations. Protein spots were in-gel digested using a trypsin digestion kit (Thermo Scientific, United States). The digested peptides were purified and concentrated using ZipTip C18 (Merck Milipore, United States) before spotting onto a target plate (AnchorChip Standard, 800 um; Bruker, United States). An UltraFlex MALDI-TOF/TOF mass spectrometer (Bruker) was used to analyze the digested peptides. Mass spectrometry spectra were gathered with 3000 laser shots per spectrum, and tandem mass spectrometry spectra were acquired with 4000 laser shots per fragmentation spectrum. The peptide mass fingerprinting peaks with the highest mass intensities (maximum 20 strongest peaks) were selected as precursor ions to acquire MS/MS fragmentation data. Bruker Daltonics Bio tools 3.2 SR3 was used for spectra analyses and the generation of peak list files. The signal-to-noise threshold was set at 7. The peak list files were used to search an in-house *B. lehensis* G1 database (4017 sequences; 1166855 residues) using MASCOT version 2.4 (Matrix Science). The search parameters were set for proteolytic enzymes: trypsin, one maximum missed cleavage, variable modification of oxidation (Methionine), fixed modification of cys residues carbamidomethylation and peptide mass tolerance for monoisotopic data of 100 ppm, and a fragment mass tolerance of 0.4 Da.

### 2.3 In silico analysis

Identified proteins were classified into functional groups using the information available in SubtiList webserver (http://genolist.pasteur.fr/SubtiList/).
