# Supplementary material for: Data for proteome analysis of Bacillus lehensis G1 in starch-containing medium
Source: Data Brief. 2017 Jul 14;14:35–40. doi: 10.1016/j.dib.2017.07.026 (PMC5524313; doi:10.1016/j.dib.2017.07.026)
Supplement: Supplementary file 1 — Supplementary material [file mmc1.docx]

Supplementary information table of proteins identified by peptide fragment fingerprinting (PFF)

| Spot no.^a^ | Gene no.^b^ | Annotation^c^ | Identified MS/MS peptide sequences | ppm | Ion score | Expect | Sequence Coverage (%) | Protein score |
| --- | --- | --- | --- | --- | --- | --- | --- | --- |
| 1 | AIC94431 | Hypothetical protein, conserved | TYDGFIDR | 11.2 | 29 | 0.0051 | 17 | 262 |
|  |  |  | LGNGVTEGPANIR | 17.9 | 31 | 0.0026 |  |  |
|  |  |  | APQTGYLWYDR | 15.0 | 38 | 0.00056 |  |  |
|  |  |  | LNETVQANLGSIR | 22.0 | 34 | 0.0013 |  |  |
|  |  |  | ADGSWPNGYGYQSVGTLR | 8.18 | 48 | 4.4e-005 |  |  |
|  |  |  | ADGSWPTNYGYFNVGDVTANQQGR | 3.61 | 48 | 3e-005 |  |  |
|  |  |  | AIEQDAIASFAENGGGVFFIADHYNADR | 5.49 | 33 | 0.0013 |  |  |
| 2 | AIC95833 | Minor extracellular protease | TGAISFGPVSTATGNIR | -15.26 | 84 | 1.7e-008 | 9 | 221 |
|  |  |  | IGVLDTGVDYNHPDLK | -13.45 | 39 | 0.00044 |  |  |
|  |  |  | VLGPYGSGATSGVIAGIER | -13.16 | 39 | 0.00046 |  |  |
|  |  |  | VIAESFETDLASLTGETLPVVSASLGR | -28.46 | 59 | 3.5e-006 |  |  |
| 3 | AIC94728 | Aconitate hydratase | RPQDLIELPK | 5.31 | 33 | 0.0021 | 24 | 482 |
|  |  |  | DIWPSTEEVR | 3.02 | 23 | 0.023 |  |  |
|  |  |  | DPEAVKPLSDLR | 3.71 | 60 | 5e-006 |  |  |
|  |  |  | EYQDVFTSNDR | 2.99 | 41 | 0.00021 |  |  |
|  |  |  | FDSDVDVDYYR | 3.34 | 38 | 0.00032 |  |  |
|  |  |  | TYHYYALDALEK | 1.74 | 39 | 0.00036 |  |  |
|  |  |  | TIVDQTVTPELFR | 2.80 | 36 | 0.001 |  |  |
|  |  |  | ESISVAIDDTVKPR | 8.01 | 12 | 0.22 |  |  |
|  |  |  | FGTDDSLLYNMNR | 5.12 | 26 | 0.0087 |  |  |
|  |  |  | KASELGLQVPEYVK | 3.45 | 19 | 0.046 |  |  |
|  |  |  | FGDSVTTDHISPAGAIGK | 3.26 | 13 | 0.16 |  |  |
|  |  |  | FVEYFGPGLADMPLADR | 1.88 | 30 | 0.0032 |  |  |
|  |  |  | VILQDFTGVPAVVDLAALR | 4.27 | 61 | 2.1e-006 |  |  |
|  |  |  | YKENDTGLVVLAGQDYGMGSSR+ Oxidation (M) | -2.23 | 19 | 0.033 |  |  |
|  |  |  | DYVTVTAVAEDGTKTEFEALVR | 2.64 | 35 | 0.00083 |  |  |
| 6 | AIC95721 | 60 kDa chaperonin | GFSTELEVVEGMQFDR | -17.80 | 7 | 0.67 | 10 | 57 |
|  |  |  | AVQAVEAAGDEATGVNIVLR | -6.06 | 41 | 0.00026 |  |  |
|  |  |  | TNDIAGDGTTTATVLAQAMIR+ Oxidation (M) | -3.66 | 9 | 0.46 |  |  |
| 10 | AIC95559 | Enolase | YNQLLR | 8.14 | 27 | 0.011 | 36 | 393 |
|  |  |  | TIISDIYAR | 1.37 | 41 | 0.00034 |  |  |
|  |  |  | AGYTAVISHR | 2.15 | 28 | 0.0078 |  |  |
|  |  |  | VQLVGDDLFVTNTK | -4.54 | 36 | 0.00094 |  |  |
|  |  |  | IEDELADLAQYNGLK | -7.57 | 75 | 1.3e-007 |  |  |
|  |  |  | ALVPSGASTGEYEAVELR | -2.65 | 30 | 0.0032 |  |  |
|  |  |  | GNPTVEVEVHLESGVMGR | -1.16 | 9 | 0.39 |  |  |
|  |  |  | SGETEDATIADIAVATNAGQIK | -4.37 | 40 | 0.00027 |  |  |
|  |  |  | YPIISIEDGLDENDWDGHK | -3.75 | 30 | 0.0024 |  |  |
|  |  |  | AVANVNETIAPELIGENVLDQIGIDR | -16.90 | 77 | 4.2e-008 |  |  |
|  |  |  | IEESGQAANELYDQQDR | -11.32 | 55 | 1.1e-005 |  |  |
|  |  |  | EGTEATINDLLEQIAINNR | -11.51 | 33 | 0.0016 |  |  |
|  |  |  | EREGTEATINDLLEQIAINNR | -14.58 | 16 | 0.075 |  |  |
|  |  |  | QQLMSEVNELVPNFFDVSTGR+ Oxidation (M) | -16.92 | 34 | 0.001 |  |  |
| 12 | AIC96376 | Hypothetical protein, conserved | GTAYWNGFHQR | -41.12 | 25 | 0.0076 | 29 | 188 |
|  |  |  | VGIYTDDGTGRPK | -21.70 | 14 | 0.21 |  |  |
|  |  |  | RDFYASPWVVTG | -32.97 | 19 | 0.045 |  |  |
|  |  |  | NWLDQPVPVLYR | -36.30 | 46 | 0.00012 |  |  |
|  |  |  | SNASLTPVSDQTDPTLR | -32.55 | 21 | 0.029 |  |  |
|  |  |  | NLAERPNPHTVFGGYR | -27.38 | 30 | 0.0033 |  |  |
|  |  |  | ATNIPYGQMPPSFPESGELFR | -41.83 | 33 | 0.0011 |  |  |
|  |  |  | ATNIPYGQMPPSFPESGELFR+ Oxidation (M) | -32.53 | 31 | 0.0019 |  |  |
| 15 | AIC93661 | Alanine dehydrogenase | VAMTPAGVVALTK+ Oxidation (M) | -6.36 | 47 | 0.00011 | 34 | 335 |
|  |  |  | MASQIGAQFLEK+ Oxidation (M) | -8.88 | 34 | 0.0018 |  |  |
|  |  |  | EPLSSEYGYFR | -3.31 | 16 | 0.09 |  |  |
|  |  |  | EVWAQSDMIMK+ 2 Oxidation (M) | -7.47 | 19 | 0.031 |  |  |
|  |  |  | DSDLVIGAVLIPGAK | -11.85 | 19 | 0.048 |  |  |
|  |  |  | TLPLLTPMSEVAGR | -7.38 | 37 | 0.00068 |  |  |
|  |  |  | TLPLLTPMSEVAGR+ Oxidation (M) | -4.78 | 45 | 0.0001 |  |  |
|  |  |  | DLGYELVSVADALAK | -11.02 | 34 | 0.0013 |  |  |
|  |  |  | VKEPLSSEYGYFR | -6.13 | 38 | 0.00082 |  |  |
|  |  |  | HGVVHYAVANMPGAVPR | -9.79 | 55 | 1e-005 |  |  |
|  |  |  | HGVVHYAVANMPGAVPR+ Oxidation (M) | -6.87 | 50 | 3.4e-005 |  |  |
|  |  |  | TSTLGLTNVTIPYAMQIANK+ Oxidation (M) | -19.24 | 30 | 0.0032 |  |  |
| 17 | AIC93909 | Sugar ABC transporter ATP-binding protein | DFVINNQR | -10.51 | 24 | 0.022 | 6 | 97 |
|  |  |  | AHFFNPDNEQR | -22.99 | 39 | 0.00034 |  |  |
|  |  |  | LDGKDFVINNQR | -22.63 | 34 | 0.002 |  |  |
| 19 | AIC96117 | Flagellin | EVDALAEEITR | 5.89 | 50 | 4.9e-005 | 26 | 219 |
|  |  |  | MIINNNLSAMNAHR | -1.19 | 40 | 0.00031 |  |  |
|  |  |  | MIINNNLSAMNAHR+ Oxidation (M) | 3.79 | 35 | 0.0012 |  |  |
|  |  |  | MIINNNLSAMNAHR+ 2 Oxidation (M) | 6.78 | 18 | 0.055 |  |  |
|  |  |  | AEIQKEVDALAEEITR | -5.64 | 31 | 0.0027 |  |  |
|  |  |  | LEHTINNLDNASENLSAAESR | -14.09 | 50 | 2.6e-005 |  |  |
|  |  |  | NAQDGISMIQTAEGALNETHSILQR | 1.47 | 11 | 0.15 |  |  |
|  |  |  | NAQDGISMIQTAEGALNETHSILQR+ Oxidation (M) | 10.2 | 48 | 3.5e-005 |  |  |
| 20 | AIC96630 | Cysteine synthase | LEYQNPGSSVK | 7.33 | 37 | 0.0009 | 31 | 254 |
|  |  |  | KVVAIIPSNGER | -3.97 | 14 | 0.12 |  |  |
|  |  |  | VSTDEAFEYAR | 2.13 | 36 | 0.0011 |  |  |
|  |  |  | LVMPETMSLER+ 2 Oxidation (M) | 10.4 | 26 | 0.0091 |  |  |
|  |  |  | LTSEEHADVYLK | 13.2 | 40 | 0.00044 |  |  |
|  |  |  | AYGADLVLTPGPEGMGGAIR | -3.04 | 37 | 0.00066 |  |  |
|  |  |  | AYGADLVLTPGPEGMGGAIR+ Oxidation (M) | -0.07 | 59 | 4.2e-006 |  |  |
|  |  |  | IVAIEPQDSPVLSGGKPGPHK | -1.32 | 42 | 0.00017 |  |  |
| 23 | AIC94426 | Hypothetical protein, conserved | ISPLYVQVTDNR | 25.0 | 38 | 0.00073 | 28 | 198 |
|  |  |  | ITSTTETYYAAPQTFR | 18.6 | 20 | 0.045 |  |  |
|  |  |  | SIAASSKPSHAQESFTLTPEAK | 9.82 | 38 | 0.00048 |  |  |
|  |  |  | NYSTTLTWTLSDTPGNEDINR | 3.70 | 80 | 2.6e-008 |  |  |
|  |  |  | NYSTTLTWTLSDTPGNEDINRK | -4.14 | 23 | 0.011 |  |  |
| 24 | AIC94046 | Chaperone protein DnaK | FQLNDIPPAPR | 9.19 | 51 | 3.3e-005 | 1 | 51 |
| 26 | AIC95608 | Flagellar hook-associated protein | ANVTAFFDEHQK | 20.1 | 35 | 0.0015 | 5 | 130 |
|  |  |  | HILIQGQAYEIVSDQAGELAR | 14.2 | 96 | 6.6e-010 |  |  |
| 28 | AIC96289 | Fructose-bisphosphate aldolase | SPVILGVSEGAAR | -27.31 | 11 | 0.32 | 12 | 56 |
|  |  |  | GEPNLGFDHMK+ Oxidation (M) | -27.78 | 7 | 0.48 |  |  |
|  |  |  | ETLAAQPEQYDPR | -31.73 | 38 | 0.00043 |  |  |
| 30 | AIC94052 | Deoxyribose-phosphate aldolase | QQILHLCEEAK | -5.26 | 35 | 0.0013 | 42 | 259 |
|  |  |  | TAAQVLAEEPEVK | -8.10 | 39 | 0.00055 |  |  |
|  |  |  | FASVCIQPYWVK | -11.06 | 22 | 0.029 |  |  |
|  |  |  | AESVAHLIDHTLLK | -4.22 | 26 | 0.011 |  |  |
|  |  |  | SFEDVQAMVEAGATR | -9.12 | 68 | 5.4e-007 |  |  |
|  |  |  | SFEDVQAMVEAGATR+ Oxidation (M) | -6.50 | 38 | 0.00054 |  |  |
|  |  |  | TSTGFSTNGATVEDIR | -5.79 | 42 | 0.00018 |  |  |
|  |  |  | VIIETSLLTNEEKER | -4.01 | 27 | 0.006 |  |  |
| 31 | AIC94978 | Dihydrolipoyllysine-residue acetyltransferase component of pyruvate dehydrogenase complex | RLLNDPQLLLMEG+ Oxidation (M) | -15.16 | 46 | 0.00011 | 25 | 498 |
|  |  |  | LIDGVTAQSALNQIK | -13.17 | 69 | 4e-007 |  |  |
|  |  |  | SIFQLADEINELAVK | -21.69 | 78 | 7.4e-008 |  |  |
|  |  |  | LIDGVTAQSALNQIKR | -15.54 | 64 | 1.5e-006 |  |  |
|  |  |  | SAKPTTSAPAASSEQLEER | -18.13 | 68 | 4.1e-007 |  |  |
|  |  |  | KYPALNASIDDEAGEIVYK | -28.58 | 47 | 5.4e-005 |  |  |
|  |  |  | QTAPHVTHLDEIDVTALVAHR | -23.85 | 65 | 7.6e-007 |  |  |
|  |  |  | SAKPTTSAPAASSEQLEERVPLK | -21.55 | 29 | 0.0027 |  |  |
|  |  |  | QTAPHVTHLDEIDVTALVAHRK | -24.21 | 32 | 0.0015 |  |  |
| 32 | AIC95922 | GlcNAc-binding protein A | NQFELVPFYEK | 31.8 | 15 | 0.17 | 18 | 80 |
|  |  |  | VNTDCGGVIYEPQSLEAPK | 17.4 | 37 | 0.0006 |  |  |
|  |  |  | WHYYITKPNWNPNQPLTR | 13.1 | 29 | 0.0034 |  |  |
| 33 | AIC92898 | Alkyl hydroperoxide reductase subunit | QVQPFSAQAYR | -26.10 | 38 | 0.00069 | 21 | 136 |
|  |  |  | DFQVLNEASGLADR | -39.64 | 63 | 1.5e-006 |  |  |
|  |  |  | ITYTMIGDPSQAISR | -51.11 | 25 | 0.014 |  |  |
|  |  |  | ITYTMIGDPSQAISR+ Oxidation (M) | -49.95 | 35 | 0.001 |  |  |
| 35 | AIC94804 | Ribosome recycling factor | IIIPALTEER | 24.9 | 15 | 0.046 | 13 | 60 |
|  |  |  | ADLGLTPSSDGQVIR | 25.1 | 45 | 0.00013 |  |  |
| 38 | AIC93828 | Phage major tail protein | EFIEIYR | 22.7 | 21 | 0.035 | 23 | 117 |
|  |  |  | LDNKEFIEIYR | 23.5 | 36 | 0.0011 |  |  |
|  |  |  | EYKGEEFIFAVK | 21.9 | 18 | 0.061 |  |  |
|  |  |  | DSQGLLRPFNQTGGSFNR | 17.8 | 43 | 0.00014 |  |  |
| 68 | AIC95525 | Cysteine desulfurase | AKEYFSDVFS | 5.57 | 22 | 0.029 | 17 | 91 |
|  |  |  | LGGPTGIGALYGK | 8.09 | 28 | 0.0085 |  |  |
|  |  |  | AFLNANDTAEVIFTR | 6.45 | 14 | 0.16 |  |  |
|  |  |  | NGAIMVVDGAQSAPHIK+ Oxidation (M) | -1.49 | 16 | 0.1 |  |  |
|  |  |  | GVHTLGTMATDEYEGAR | 4.91 | 6 | 0.67 |  |  |
|  |  |  | GVHTLGTMATDEYEGAR+ Oxidation (M) | 1.99 | 11 | 0.25 |  |  |
| 69 | AIC94431 | Hypothetical protein, conserved | TYDGFIDR | -1.88 | 31 | 0.0029 | 8 | 92 |
|  |  |  | LGNGVTEGPANIR | -1.08 | 25 | 0.01 |  |  |
|  |  |  | APQTGYLWYDR | -0.76 | 24 | 0.013 |  |  |
|  |  |  | AIEQDAIASFAENGGGVFFIADHYNADR | -6.20 | 13 | 0.13 |  |  |
| 70 | AIC95782 | Sulfatase | VIYGDLLR | 51.0 | 12 | 0.2 | 11 | 115 |
|  |  |  | EEITPFDTIQLQR | 35.6 | 24 | 0.016 |  |  |
|  |  |  | EDIQFGSDLFAPER | 36.0 | 42 | 0.0002 |  |  |
|  |  |  | TSDSEFIVANSMFGR+ Oxidation (M) | 37.4 | 12 | 0.26 |  |  |
|  |  |  | ETGEPIEDEEGVDACGEYAER | 21.7 | 26 | 0.005 |  |  |
| 71 | AIC93540 | Chitinase | NTFAQSAVDFVR | 7.24 | 55 | 1.1e-005 | 12 | 172 |
|  |  |  | TVISVGGWTWSNR | 3.27 | 8 | 0.68 |  |  |
|  |  |  | GLGGAMFWEASNNR | 11.4 | 46 | 9.4e-005 |  |  |
|  |  |  | GLGGAMFWEASNNR+ Oxidation (M) | 10.9 | 13 | 0.16 |  |  |
|  |  |  | VVGYYPSWVAEER | 7.78 | 36 | 0.00096 |  |  |
|  |  |  | GNINQLNQLKEEYPHLK | -1.74 | 27 | 0.0068 |  |  |
| 73 | AIC96260 | ATP synthase subunit alpha | HVLVIYDDLSK | -2.85 | 20 | 0.036 | 12 | 78 |
|  |  |  | EAFPGDVFYLHSR | -2.28 | 10 | 0.42 |  |  |
|  |  |  | VVNSLGQPIDGLGPIHTTK | -8.20 | 23 | 0.016 |  |  |
|  |  |  | ELEAFSQFGSDLDAATQAR | -7.03 | 26 | 0.0057 |  |  |
| 74 | AIC96258 | ATP synthase subunit beta | YDDLPEDAFR | 20.5 | 21 | 0.03 | 73 | 172 |
|  |  |  | FTQAGSEVSALLGR | 15.9 | 12 | 0.21 |  |  |
|  |  |  | VALSGLTMAEYFR | 16.2 | 11 | 0.35 |  |  |
|  |  |  | FPSGQLPQINSALR | 13.9 | 39 | 0.00053 |  |  |
|  |  |  | TAMVFGQMNEPPGAR | 13.6 | 11 | 0.28 |  |  |
|  |  |  | TAMVFGQMNEPPGAR+ Oxidation (M) | 14.9 | 14 | 0.15 |  |  |
|  |  |  | ALSPEVVGEEHYSVAR | 14.3 | 42 | 0.0002 |  |  |
|  |  |  | DEQGADVLLFVDNIFR | 13.8 | 14 | 0.13 |  |  |
|  |  |  | LTEQGIYPAVDPLASTSR | 11.2 | 9 | 0.44 |  |  |
|  |  |  | KLTEQGIYPAVDPLASTSR | 8.92 | 48 | 3.8e-005 |  |  |
|  |  |  | GTEAVDTGAPISVPVGEETLGR | 6.75 | 15 | 0.08 |  |  |
|  |  |  | VFNVLGEEIDLKEPVPAGTR | 11.1 | 11 | 0.2 |  |  |
|  |  |  | MPSAVGYQPTLATEMGQLQER+ Oxidation (M) | 9.44 | 9 | 0.3 |  |  |
|  |  |  | MPSAVGYQPTLATEMGQLQER+ 2 Oxidation (M) | 9.31 | 14 | 0.089 |  |  |
| 75 | AIC95608 | Flagellar hook-associated protein | ANVTAFFDEHQK | -14.70 | 27 | 0.0087 | 4 | 62 |
|  |  |  | NDSLVQTSLNQLR | -13.51 | 35 | 0.0014 |  |  |
| 76 | AIC95608 | Flagellar hook-associated protein | ANVTAFFDEHQK | -8.82 | 19 | 0.047 | 7 | 114 |
|  |  |  | NDSLVQTSLNQLR | -8.93 | 21 | 0.037 |  |  |
|  |  |  | HILIQGQAYEIVSDQAGELAR | -13.25 | 73 | 1.1e-007 |  |  |
| 78 | AIC94429 | Legume lectin, beta chain domain-containing protein | GFLGTQNAR | -30.69 | 8 | 0.98 | 7 | 67 |
|  |  |  | NITSGETAFQR | -28.59 | 19 | 0.05 |  |  |
|  |  |  | TGFFLDVLPGNAR | -23.22 | 12 | 0.28 |  |  |
|  |  |  | QIADGLTFTMHNDPR+ Oxidation (M) | -28.68 | 10 | 0.35 |  |  |
|  |  |  | GLQNQSHTAVVNPSVIDR | -30.26 | 18 | 0.046 |  |  |
| 80 | AIC95481 | Cytosol aminopeptidase | HALGNVGLK | -15.14 | 45 | 0.00017 | 17 | 222 |
|  |  |  | TLAWFVAHS | -22.32 | 33 | 0.0026 |  |  |
|  |  |  | QGELHKDELR | -24.95 | 49 | 5.8e-005 |  |  |
|  |  |  | GEFTQGVQAIDR | -26.96 | 29 | 0.0058 |  |  |
|  |  |  | TSDVADLNNAPGR | -23.78 | 10 | 0.33 |  |  |
|  |  |  | DKGSSLGPAGATGIMAR | -28.20 | 6 | 1 |  |  |
|  |  |  | DKGSSLGPAGATGIMAR+ Oxidation (M) | -29.35 | 19 | 0.054 |  |  |
|  |  |  | QAELGYTYGEGANTAR | -28.60 | 38 | 0.00043 |  |  |
| 81 | AIC94131 | Fumarate hydratase class II | YWGAQTQR | -52.45 | 20 | 0.029 | 4 | 29 |
|  |  |  | DTIGEIQVPADKYWGAQTQR | -45.22 | 9 | 0.35 |  |  |
| 86 | AIC96376 | Hypothetical protein, conserved | FNGEVFLR | -11.25 | 35 | 0.0018 | 10 | 70 |
|  |  |  | GTAYWNGFHQR | -15.87 | 25 | 0.011 |  |  |
|  |  |  | SNASLTPVSDQTDPTLR | -14.85 | 11 | 0.34 |  |  |
| 92 | AIC96288 | Translaldolase | AGATYVSPFLGR | -11.51 | 52 | 2.1e-005 | 59 | 357 |
|  |  |  | ELAAIAENITVK | -14.05 | 19 | 0.055 |  |  |
|  |  |  | GIEQFLSDWNK | -15.81 | 37 | 0.00089 |  |  |
|  |  |  | FFIDTANLSEIK | -14.96 | 42 | 0.00027 |  |  |
|  |  |  | LDDIGHDGLNLITDVR | -22.00 | 53 | 1.8e-005 |  |  |
|  |  |  | TIFDTHGLDTQIIAASVR | -27.19 | 72 | 2e-007 |  |  |
|  |  |  | EAHELGILDGVTTNPTLVAK | -29.54 | 55 | 6.4e-006 |  |  |
|  |  |  | EIAAVVPGSVSAEVISLEAEEMIR | -32.05 | 28 | 0.0035 |  |  |
| 96 | AIC96376 | Hypothetical protein, conserved | GTAYWNGFHQR | -5.04 | 37 | 0.00077 | 13 | 68 |
|  |  |  | NLAERPNPHTVFGGYR | -4.76 | 8 | 0.56 |  |  |
|  |  |  | ATNIPYGQMPPSFPESGELFR+ Oxidation (M) | -14.60 | 23 | 0.013 |  |  |
| 98 | AIC95922 | GlcNAc-binding protein A | NQFELVPFYEK | -18.11 | 40 | 0.00052 | 18 | 92 |
|  |  |  | VNTDCGGVIYEPQSLEAPK | -60.66 | 39 | 0.00019 |  |  |
|  |  |  | WHYYITKPNWNPNQPLTR | -71.85 | 13 | 0.11 |  |  |
| 101 | AIC94216 | 2-methylcitrate dehydratase | DYLDPNKR | -11.99 | 7 | 1.1 | 17 | 105 |
|  |  |  | GAFNIGTMIR | -10.53 | 5 | 1.4 |  |  |
|  |  |  | VPGTSHVLDPIR | -1.44 | 25 | 0.015 |  |  |
|  |  |  | AHEIQGVLALDNSFNR | -2.27 | 21 | 0.033 |  |  |
|  |  |  | DGSSTDQIAVEYPLGHR | -4.28 | 16 | 0.07 |  |  |
|  |  |  | GDIVADDYEDDMANDPR | -5.81 | 6 | 0.38 |  |  |
|  |  |  | GDIVADDYEDDMANDPR+ Oxidation (M) | -8.08 | 30 | 0.0011 |  |  |
| 102 | AIC95918 | Trifunctional nucleotide phosphoesterase protein | SGQWDVWK | -3.25 | 11 | 0.38 | 9 | 138 |
|  |  |  | WWTQGEDPR | 0.20 | 18 | 0.062 |  |  |
|  |  |  | IMEVLVEQSR | 3.85 | 15 | 0.16 |  |  |
|  |  |  | WWTQGEDPRR | 3.79 | 10 | 0.42 |  |  |
|  |  |  | MLPVQLVNGTAVR | 5.20 | 6 | 1.2 |  |  |
|  |  |  | SSITGEIPPGSITDR | 5.62 | 32 | 0.0024 |  |  |
|  |  |  | TVAEAIGNNSGVYPVK | 6.75 | 19 | 0.054 |  |  |
|  |  |  | STQDVGIGNLYTDAIR | 6.21 | 28 | 0.005 |  |  |
| 103 | AIC95608 | Flagellar hook-associated protein | ANVTAFFDEHQK | -24.17 | 40 | 0.00038 | 7 | 70 |
|  |  |  | NDSLVQTSLNQLR | -20.02 | 23 | 0.023 |  |  |
|  |  |  | HILIQGQAYEIVSDQAGELAR | -32.62 | 7 | 0.51 |  |  |
| 104 | AIC95220 | Succinate dehydrogenase flavoprotein subunit | VITIGAYNR | 8.54 | 6 | 1.1 | 5 | 47 |
|  |  |  | YPAYGNLVPR | 9.21 | 21 | 0.037 |  |  |
|  |  |  | GLTAQELNSAEIR | 9.57 | 20 | 0.042 |  |  |
| 105 | AIC96492 | Cyclomaltodextrin glucanotransferase | DVIYQVVTDR | 12.4 | 40 | 0.00041 | 23 | 339 |
|  |  |  | QTDMALAVLLTSR | 12.7 | 29 | 0.0053 |  |  |
|  |  |  | QTDMALAVLLTSR+ Oxidation (M) | 15.4 | 13 | 0.21 |  |  |
|  |  |  | EFEVLSGNQVSVR | 15.5 | 42 | 0.0003 |  |  |
|  |  |  | QTNSALGYGTTTER | 18.0 | 20 | 0.036 |  |  |
|  |  |  | WLNEDIYIYER | 16.6 | 42 | 0.00024 |  |  |
|  |  |  | DQNGNVVWQSGNNR | 11.9 | 45 | 9.9e-005 |  |  |
|  |  |  | TNPYYGNFDDFDR | 20.6 | 27 | 0.0055 |  |  |
|  |  |  | SGNTITVSGEGFGDER | 22.0 | 9 | 0.44 |  |  |
|  |  |  | LDGNTITVNANGAVNSFQLR | 8.46 | 29 | 0.0038 |  |  |
|  |  |  | EYNEVIDQVTFIDNHDMSR | 6.65 | 30 | 0.003 |  |  |
|  |  |  | EYNEVIDQVTFIDNHDMSR+ Oxidation (M) | 7.80 | 12 | 0.13 |  |  |
|  |  |  | GVPTIYYGTEQYVTGGNDPENR | 6.17 | 26 | 0.006 |  |  |
| 106 | AIC96492 | Cyclomaltodextrin glucanotransferase | DVIYQVVTDR | 23.3 | 39 | 0.00059 | 18 | 301 |
|  |  |  | QTDMALAVLLTSR | 20.8 | 25 | 0.014 |  |  |
|  |  |  | QTDMALAVLLTSR+ Oxidation (M) | 24.5 | 17 | 0.069 |  |  |
|  |  |  | EFEVLSGNQVSVR | 22.7 | 22 | 0.029 |  |  |
|  |  |  | QTNSALGYGTTTER | 27.6 | 22 | 0.021 |  |  |
|  |  |  | WLNEDIYIYER | 21.4 | 41 | 0.00034 |  |  |
|  |  |  | DQNGNVVWQSGNNR | 16.1 | 47 | 6e-005 |  |  |
|  |  |  | TNPYYGNFDDFDR | 26.3 | 22 | 0.018 |  |  |
|  |  |  | KTNPYYGNFDDFDR | 25.9 | 10 | 0.34 |  |  |
|  |  |  | EYNEVIDQVTFIDNHDMSR | 9.23 | 49 | 3.5e-005 |  |  |
|  |  |  | EYNEVIDQVTFIDNHDMSR+ Oxidation (M) | 10.8 | 16 | 0.047 |  |  |
|  |  |  | GVPTIYYGTEQYVTGGNDPENR | 7.36 | 23 | 0.01 |  |  |
| 107 | AIC96492 | Cyclomaltodextrin glucanotransferase | DVIYQVVTDR | -0.23 | 37 | 0.00095 | 18 | 241 |
|  |  |  | QTDMALAVLLTSR | -3.41 | 14 | 0.2 |  |  |
|  |  |  | EFEVLSGNQVSVR | -0.40 | 30 | 0.0047 |  |  |
|  |  |  | QTNSALGYGTTTER | 1.23 | 20 | 0.032 |  |  |
|  |  |  | WLNEDIYIYER | -0.80 | 42 | 0.00024 |  |  |
|  |  |  | DQNGNVVWQSGNNR | -5.61 | 36 | 0.0007 |  |  |
|  |  |  | TNPYYGNFDDFDR | -9.22 | 23 | 0.01 |  |  |
|  |  |  | EYNEVIDQVTFIDNHDMSR | -11.40 | 16 | 0.058 |  |  |
|  |  |  | EYNEVIDQVTFIDNHDMSR+ Oxidation (M) | -10.97 | 15 | 0.058 |  |  |
|  |  |  | GVPTIYYGTEQYVTGGNDPENR | -9.52 | 24 | 0.0094 |  |  |
| 108 | AIC93567 | Heat shock protein Hsp90 | FYQAFGR | -12.35 | 9 | 0.59 | 13 | 279 |
|  |  |  | LVSLADYVSR | -23.08 | 45 | 0.00017 |  |  |
|  |  |  | YDKFYQAFGR | -28.85 | 36 | 0.0011 |  |  |
|  |  |  | SAELVPDYFSFVK | -29.33 | 48 | 6.6e-005 |  |  |
|  |  |  | EYDGKEFISVSSADLK | -30.24 | 60 | 4.5e-006 |  |  |
|  |  |  | SELTDEDYVQFYQDKR | -33.76 | 50 | 2.3e-005 |  |  |
|  |  |  | MKEDQTHIYYATGDSVER | -37.80 | 32 | 0.0013 |  |  |
|  |  |  | MKEDQTHIYYATGDSVER+ Oxidation (M) | -35.73 | 20 | 0.022 |  |  |
| 109 | AIC96089 | Hypothetical protein, conserved | FYLNEVDINLR | -9.78 | 35 | 0.0012 | 10 | 91 |
|  |  |  | LHSIELEKNEMR | -9.38 | 25 | 0.012 |  |  |
|  |  |  | LHSIELEKNEMR+ Oxidation (M) | -2.55 | 14 | 0.17 |  |  |
|  |  |  | YAESGEWYVHSISVGDK | -11.43 | 31 | 0.0021 |  |  |
| 110 | AIC95790 | Xylose isomerase | LIPYWK | 38.9 | 12 | 0.19 | 35 | 298 |
|  |  |  | WQWEEK | 24.4 | 31 | 0.0041 |  |  |
|  |  |  | TWSDIISALR | 17.1 | 25 | 0.016 |  |  |
|  |  |  | QDAVHFFHAK | 20.7 | 32 | 0.0027 |  |  |
|  |  |  | ASHESFVQTVK | 25.1 | 29 | 0.0055 |  |  |
|  |  |  | EMAAFAEEHGVK+ Oxidation (M) | 28.7 | 30 | 0.0046 |  |  |
|  |  |  | AVSNLQQVLMKEPAPK+ Oxidation (M) | 13.1 | 54 | 1.4e-005 |  |  |
|  |  |  | ALQVPTVTTFSGTPGAYEGDK | 4.91 | 64 | 1.1e-006 |  |  |
|  |  |  | GLTISSLSCHGNALSPDEAFAK | 11.6 | 21 | 0.026 |  |  |
| 111 | AIC92706 | Endonuclease/CDSuclease/phosphatase | SILAESIEINDYR | -8.07 | 45 | 0.00011 | 21 | 176 |
|  |  |  | NSEGNDPSPHSWNER | -12.02 | 50 | 1.9e-005 |  |  |
|  |  |  | LGTFNGFNDPTGGGADNK | -13.67 | 25 | 0.012 |  |  |
|  |  |  | NGEQYPSDHFPVIAEFK | -17.29 | 56 | 6.8e-006 |  |  |
| 112 | AIC95608 | Flagellar hook-associated protein | ANVTAFFDEHQK | -25.03 | 44 | 0.00015 | 14 | 271 |
|  |  |  | DIATNEMEWVVGK+ Oxidation (M) | -29.12 | 38 | 0.00055 |  |  |
|  |  |  | DTTNQAITLAASTDVDSIYK | -26.23 | 56 | 8.5e-006 |  |  |
|  |  |  | EFTFEGSLFTSTFGLNEVK | -32.90 | 36 | 0.00077 |  |  |
|  |  |  | HILIQGQAYEIVSDQAGELAR | -30.49 | 99 | 2.7e-010 |  |  |
| 115 | AIC95591 | Hypothetical protein, conserved | LFVADDVR | -6.31 | 31 | 0.0051 | 27 | 321 |
|  |  |  | AVANTGSITLR | -21.25 | 30 | 0.0049 |  |  |
|  |  |  | LFVADDVRFEGK | -27.94 | 14 | 0.16 |  |  |
|  |  |  | YMHVTANDTLPGK+ Oxidation (M) | -30.45 | 10 | 0.38 |  |  |
|  |  |  | TNNAEQAHDFLLK | -32.70 | 46 | 6.9e-005 |  |  |
|  |  |  | LYTFNGSLTGESLK | -34.19 | 53 | 2e-005 |  |  |
|  |  |  | VNGTLYVPTSTYDSIK | -41.57 | 62 | 2e-006 |  |  |
|  |  |  | SNVGSIHNHLESSVIHDEK | -39.67 | 39 | 0.00033 |  |  |
|  |  |  | SNVGSIHNHLESSVIHDEKK | -41.75 | 37 | 0.00064 |  |  |
| 116 | AIC96453 | Purine nucleoside phosphorylase deoD-type | NMLGFTGTYK+ Oxidation (M) | 3.67 | 10 | 0.38 | 21 | 81 |
|  |  |  | FIAETFLEDVEQFNSVR | -7.66 | 23 | 0.013 |  |  |
|  |  |  | QTAHINPTGEIAETVLLPGDPLR | -24.04 | 48 | 3.7e-005 |  |  |
| 117 | AIC95591 | Hypothetical protein, conserved | LFVADDVRFEGK | 2.57 | 23 | 0.021 | 16 | 182 |
|  |  |  | TNNAEQAHDFLLK | 3.52 | 48 | 5.1e-005 |  |  |
|  |  |  | LYTFNGSLTGESLK | 0.40 | 61 | 3e-006 |  |  |
|  |  |  | SNVGSIHNHLESSVIHDEK | 4.82 | 28 | 0.0044 |  |  |
|  |  |  | SNVGSIHNHLESSVIHDEKK | 2.96 | 22 | 0.021 |  |  |
| 118 | AIC94116 | Glucokinase | AGEDLLAPLR | 8.00 | 24 | 0.013 | 6 | 66 |
|  |  |  | VVFDQYALPR | -5.12 | 43 | 0.00024 |  |  |
| 120 | AIC93828 | Phage major tail protein | EFIEIYR | 11.9 | 26 | 0.012 | 23 | 120 |
|  |  |  | LDNKEFIEIYR | 8.66 | 38 | 0.00062 |  |  |
|  |  |  | EYKGEEFIFAVK | 6.76 | 13 | 0.2 |  |  |
|  |  |  | DSQGLLRPFNQTGGSFNR | 4.55 | 44 | 0.00012 |  |  |
| 121 | AIC94609 | Nucleoside diphosphate kinase | NLIGPIVSR | -11.05 | 35 | 0.00094 | 33 | 73 |
|  |  |  | ETAETHYGEHR | -15.41 | 9 | 0.31 |  |  |
|  |  |  | TYVMIKPDGVQR | -17.56 | 5 | 1.3 |  |  |
|  |  |  | HLIGATNPAEATPGSIR | -15.21 | 24 | 0.016 |  |  |
| 122 | AIC96118 | Hypothetical protein, conserved | VREDFPHAQFVK | -33.35 | 12 | 0.31 | 13 | 60 |
|  |  |  | HPDFEYFDEFQFR | -22.84 | 48 | 3.1e-005 |  |  |
| 124 | AIC96238 | Endopeptidase lytE | FIHTGSSR | 9.11 | 28 | 0.009 | 6 | 136 |
|  |  |  | QNGIELPR | 10.5 | 60 | 3.9e-006 |  |  |
|  |  |  | GVEVSDLNNSYWAPR | 9.16 | 48 | 4.6e-005 |  |  |
| 125 | AIC96238 | Endopeptidase lytE | FIHTGSSR | -14.77 | 29 | 0.006 | 6 | 144 |
|  |  |  | QNGIELPR | -16.37 | 37 | 0.00065 |  |  |
|  |  |  | GVEVSDLNNSYWAPR | -6.44 | 77 | 6.1e-008 |  |  |
| 127 | AIC95945 | Cell surface protein | ANGSFSNVTVLSEDTAADKR | 7.69 | 46 | 7.8e-005 | 8 | 46 |
| 129 | AIC95044 | D-alanine aminotransferase | TENGSIYVQITR | 12.8 | 20 | 0.045 | 28 | 218 |
|  |  |  | GYHFGDGVYEVIR | 16.0 | 64 | 1.6e-006 |  |  |
|  |  |  | QEEGVAAYVTEDVR | 19.5 | 25 | 0.012 |  |  |
|  |  |  | AFDHDCEEAILYR | 17.3 | 48 | 6.4e-005 |  |  |
|  |  |  | KIELEIPHSFEGFK | 12.6 | 18 | 0.059 |  |  |
|  |  |  | KAFDHDCEEAILYR | 17.0 | 29 | 0.0047 |  |  |
|  |  |  | VYNGHYFTLDEHLTR | 11.9 | 14 | 0.11 |  |  |
| 132 | AIC96381 | Siphovirus tail component | GQLQNGLIR | -9.00 | 21 | 0.024 | 15 | 53 |
|  |  |  | AEPKPLIFHDER | -22.49 | 21 | 0.027 |  |  |
|  |  |  | LDSQYVTAYTIKPNGER | -25.14 | 10 | 0.31 |  |  |
| 133 | AIC95274 | Citrate synthase | AEYIGPDKR | -8.05 | 15 | 0.2 | 16 | 85 |
|  |  |  | IPTIVTGFER | -13.67 | 14 | 0.21 |  |  |
|  |  |  | HDLFTPIFAVSR | -13.79 | 38 | 0.0008 |  |  |
|  |  |  | AYPINDVHPMAALR | -13.94 | 8 | 0.57 |  |  |
|  |  |  | AYPINDVHPMAALR+ Oxidation (M) | -12.60 | 10 | 0.33 |  |  |
|  |  |  | ALVLHADHELNASTFTAR | -9.07 | 9 | 0.58 |  |  |
| 137 | AIC94099 | Superoxide dismutase [Mn] | DIEDLVANLDSVPENIR | 23.4 | 16 | 0.089 | 32 | 34 |
|  |  |  | HHNTYVTNLNAALEGHEDLASK | 9.38 | 8 | 0.45 |  |  |
|  |  |  | LPELPYAANALEPHIDEQTMTIHHGK+ Oxidation (M) | 24.7 | 11 | 0.17 |  |  |
| 138 | AIC96238 | Endopeptidase lytE | FIHTGSSR | 39.9 | 24 | 0.022 | 6 | 126 |
|  |  |  | QNGIELPR | 22.8 | 46 | 8.6e-005 |  |  |
|  |  |  | GVEVSDLNNSYWAPR | 3.64 | 56 | 8.2e-006 |  |  |
| 139 | AIC92651 | Elongation factor G | VYSGTLNSGSYVR | -2.81 | 27 | 0.0098 | 4 | 41 |
|  |  |  | YLEGEEPTNEELVAAIR | -7.77 | 14 | 0.12 |  |  |
| 140 | AIC96258 | ATP synthase subunit beta | YDDLPEDAFR | -2.69 | 12 | 0.21 | 17 | 80 |
|  |  |  | TAMVFGQMNEPPGAR+ Oxidation (M) | -11.70 | 6 | 0.91 |  |  |
|  |  |  | ALSPEVVGEEHYSVAR | -7.31 | 25 | 0.011 |  |  |
|  |  |  | KLTEQGIYPAVDPLASTSR | -9.07 | 21 | 0.02 |  |  |
|  |  |  | GTEAVDTGAPISVPVGEETLGR | -10.05 | 16 | 0.065 |  |  |
| 142 | AIC96297 | Acetyl-CoA acetyltransferase | TIIDQDESPR | 2.01 | 37 | 0.00098 | 5 | 63 |
|  |  |  | TPLATIVCHER | -0.70 | 26 | 0.0085 |  |  |
| 143 | AIC95316 | Acetyl-CoA synthetase | VFIFMPR+ Oxidation (M) | 11.5 | 11 | 0.32 | 3 | 44 |
|  |  |  | ADQIALYYSDAR | 3.50 | 33 | 0.0016 |  |  |
| 144 | AIC93282 | Endo-beta-1,3-glucanase | FSQTYGR | 9.93 | 22 | 0.031 | 2 | 22 |
| 145 | AIC94978 | Dihydrolipoyllysine-residue acetyltransferase component of pyruvate dehydrogenase complex | LTYLPYVVK | 8.60 | 20 | 0.033 | 6 | 83 |
|  |  |  | SAKPTTSAPAASSEQLEER | 12.5 | 63 | 1.5e-006 |  |  |
| 146 | AIC94806 | Elongation factor Ts | YEVGEGIEKR | -12.07 | 24 | 0.02 | 8 | 69 |
|  |  |  | QQALNEGKPENIVEK | -5.29 | 45 | 0.00011 |  |  |
| 147 | AIC95354 | Carbonic anhydrase | QYEQFYVGK | 15.6 | 17 | 0.12 | 23 | 60 |
|  |  |  | IVILTCMDTR+ Oxidation (M) | 17.5 | 19 | 0.043 |  |  |
|  |  |  | GIKMDHIDSLR+ Oxidation (M) | 16.3 | 7 | 1 |  |  |
|  |  |  | NAGAVISHPFGSIMR+ Oxidation (M) | 12.9 | 18 | 0.077 |  |  |
| 150 | AIC96492 | Cyclomaltodextrin glucanotransferase | DVIYQVVTDR | 8.59 | 31 | 0.0034 | 21 | 405 |
|  |  |  | QTDMALAVLLTSR+ Oxidation (M) | 4.47 | 32 | 0.0027 |  |  |
|  |  |  | EFEVLSGNQVSVR | -1.65 | 37 | 0.00093 |  |  |
|  |  |  | QTNSALGYGTTTER | 1.39 | 9 | 0.42 |  |  |
|  |  |  | WLNEDIYIYER | -9.90 | 47 | 7.4e-005 |  |  |
|  |  |  | DQNGNVVWQSGNNR | -13.70 | 29 | 0.003 |  |  |
|  |  |  | TNPYYGNFDDFDR | -10.52 | 30 | 0.0018 |  |  |
|  |  |  | SGNTITVSGEGFGDER | -7.21 | 18 | 0.046 |  |  |
|  |  |  | SPTYKEFEVLSGNQVSVR | -23.18 | 75 | 1.1e-007 |  |  |
|  |  |  | EYNEVIDQVTFIDNHDMSR | -30.62 | 52 | 1.2e-005 |  |  |
|  |  |  | EYNEVIDQVTFIDNHDMSR+ Oxidation (M) | -30.20 | 38 | 0.00026 |  |  |
|  |  |  | GVPTIYYGTEQYVTGGNDPENR | -29.84 | 45 | 6.7e-005 |  |  |
| 151 | AIC96492 | Cyclomaltodextrin glucanotransferase | DVIYQVVTDR | 17.2 | 39 | 0.0006 | 19 | 481 |
|  |  |  | QTDMALAVLLTSR | 4.17 | 30 | 0.0052 |  |  |
|  |  |  | QTDMALAVLLTSR+ Oxidation (M) | 9.71 | 38 | 0.00059 |  |  |
|  |  |  | EFEVLSGNQVSVR | 7.07 | 49 | 5.3e-005 |  |  |
|  |  |  | WLNEDIYIYER | 1.26 | 49 | 4.7e-005 |  |  |
|  |  |  | DQNGNVVWQSGNNR | -2.05 | 58 | 4.3e-006 |  |  |
|  |  |  | TNPYYGNFDDFDR | 0.78 | 39 | 0.0003 |  |  |
|  |  |  | SGNTITVSGEGFGDER | 3.14 | 12 | 0.17 |  |  |
|  |  |  | SPTYKEFEVLSGNQVSVR | -12.12 | 82 | 1.9e-008 |  |  |
|  |  |  | EYNEVIDQVTFIDNHDMSR | -19.59 | 45 | 7.4e-005 |  |  |
|  |  |  | EYNEVIDQVTFIDNHDMSR+ Oxidation (M) | -20.33 | 27 | 0.0041 |  |  |
|  |  |  | GVPTIYYGTEQYVTGGNDPENR | -18.63 | 69 | 2.9e-007 |  |  |
| 155 | AIC92675 | Adenylate kinase | TYHLLYNPPK | 11.5 | 20 | 0.043 | 26 | 180 |
|  |  |  | CDVDGSELIQR | 12.3 | 40 | 0.00052 |  |  |
|  |  |  | DCENGFLLDGFPR | 8.54 | 41 | 0.00032 |  |  |
|  |  |  | TYHLLYNPPKVEGK | 10.0 | 40 | 0.00033 |  |  |
|  |  |  | RLEVNQQQAQPLIDFYESK | 6.47 | 39 | 0.00035 |  |  |
| 156 | AIC95918 | Trifunctional nucleotide phosphoesterase protein | TGSDIGLLNR | 14.6 | 41 | 0.0004 | 17 | 389 |
|  |  |  | VVGYSDTGLTR | 20.9 | 16 | 0.1 |  |  |
|  |  |  | MLPVQLVNGTAVR+ Oxidation (M) | -10.40 | 42 | 0.00029 |  |  |
|  |  |  | SSITGEIPPGSITDR | 15.7 | 49 | 4.7e-005 |  |  |
|  |  |  | DFADADVALTNNGGLR | 13.0 | 53 | 2.3e-005 |  |  |
|  |  |  | STQDVGIGNLYTDAIR | 14.2 | 43 | 0.00014 |  |  |
|  |  |  | FNGDAPLGNFWTDAMR | -7.43 | 24 | 0.013 |  |  |
|  |  |  | FNGDAPLGNFWTDAMR+ Oxidation (M) | -6.02 | 15 | 0.1 |  |  |
|  |  |  | QIEALDSFGNAIVVVETTGER | 0.19 | 22 | 0.019 |  |  |
|  |  |  | LAEAVDYFDVIIGGHSHTTLR | 0.13 | 61 | 2.7e-006 |  |  |
|  |  |  | YHNGVDLQASGLTYTLIPDGNR | -1.06 | 39 | 0.00027 |  |  |
| 157 | AIC95608 | Flagellar hook-associated protein | ANVTAFFDEHQK | 0.15 | 33 | 0.0021 | 13 | 160 |
|  |  |  | ALHTSMFDSIMR+ 2 Oxidation (M) | 2.70 | 5 | 0.93 |  |  |
|  |  |  | NDSLVQTSLNQLR | 4.15 | 44 | 0.00021 |  |  |
|  |  |  | DYDPLTDEERDGLSEAEAK | 2.17 | 8 | 0.4 |  |  |
|  |  |  | HILIQGQAYEIVSDQAGELAR | -2.07 | 71 | 2e-007 |  |  |
| 158 | AIC96475 | Endo-1,3(4)-beta-glucanase 1 | YVDLHYR | 31.4 | 26 | 0.013 | 19 | 248 |
|  |  |  | VITFTPNTPAR | 22.3 | 32 | 0.0022 |  |  |
|  |  |  | VDDYGDWHVR | 15.0 | 28 | 0.0061 |  |  |
|  |  |  | GDFTVSIEQSQSDR | 21.7 | 34 | 0.0015 |  |  |
|  |  |  | INDHHFHYGYFVK | -1.47 | 23 | 0.019 |  |  |
|  |  |  | DFAADREDPMYPYLR | -3.56 | 10 | 0.26 |  |  |
|  |  |  | DGESVYVAYNYDDEAR | 1.66 | 29 | 0.0034 |  |  |
|  |  |  | DFAADREDPMYPYLR+ Oxidation (M) | 1.34 | 6 | 0.73 |  |  |
|  |  |  | IDDYTEFDPGDETIIER | 0.76 | 16 | 0.067 |  |  |
|  |  |  | LPNTDAETLALFAEHAYSVVR | -2.51 | 11 | 0.2 |  |  |
|  |  |  | LSNLAPLADQLGEQELGEEFR | -2.80 | 17 | 0.072 |  |  |
|  |  |  | TTYSANTEAVAGGENGTLFALYPHQYR | -3.26 | 21 | 0.017 |  |  |
| 159 | AIC96380 | Phage protein | FTNPFHLK | 13.2 | 30 | 0.0055 | 29 | 398 |
|  |  |  | DHYVLSFR | 14.8 | 36 | 0.0012 |  |  |
|  |  |  | LGVVSNFPTR | 15.7 | 32 | 0.003 |  |  |
|  |  |  | LSQTVYPANR | 15.2 | 17 | 0.1 |  |  |
|  |  |  | NVSVESGLTNR | 16.2 | 17 | 0.098 |  |  |
|  |  |  | VYDKELGIDR | 15.7 | 24 | 0.017 |  |  |
|  |  |  | LPGARPVVLSPER | 17.1 | 7 | 0.61 |  |  |
|  |  |  | SAVQNFSVNENGR | 13.6 | 45 | 0.00011 |  |  |
|  |  |  | EQAQAALESLSLPR | 17.6 | 66 | 9.9e-007 |  |  |
|  |  |  | ASDLSILSGMEHEQFK+ Oxidation (M) | 12.2 | 21 | 0.026 |  |  |
|  |  |  | NIQSIEATYDTDELITR | 13.5 | 57 | 7e-006 |  |  |
|  |  |  | ENLSIDSVNDQVPYLDIR | 12.1 | 46 | 8.3e-005 |  |  |

^a^ Spot number corresponding to spots in Figure 1

^b^ The AIC gene numbering is according to the NCBI taxonomy database for strain *B. lehensis* G1.

^c^ The annotation was primarily based on the genome annotation of *B. leheniss* G1

Spot number: 6

MS/MS Peptide Sequence: AVQAVEAAGDEATGVNIVLR (Ions score: 41)

Spot number: 24

MS/MS Peptide Sequence: FQLNDIPPAPR (Ions score: 51)

Spot number: 35

MS/MS Peptide Sequence: ADLGLTPSSDGQVIR (Ions score: 45)

Spot number: 81

MS/MS Peptide Sequence: YWGAQTQR (Ions score: 20)

Spot number: 122

MS/MS Peptide Sequence: HPDFEYFDEFQFR (Ions score: 48)

Spot number: 127

MS/MS Peptide Sequence: ANGSFSNVTVLSEDTAADKR (Ions score: 46)

Spot number: 139

MS/MS Peptide Sequence: VYSGTLNSGSYVR (Ions score: 27)

Spot number: 144

MS/MS Peptide Sequence: FSQTYGR (Ions score: 22)

Supplementary information table of proteins identified by peptide mass fingerprinting (PMF)

| Spot no.^a^ | Gene no.^b^ | Annotation^c^ | Identified MS peptide sequences | ppm | Start-End | Matched MS peaks | Sequence Coverage (%) | Protein score |
| --- | --- | --- | --- | --- | --- | --- | --- | --- |
| 11 | AIC95613 | Flagella hook-associated protein 1 | VGTGVGIDR | 15.2 | 64 -72 | 23/86 | 44 | 119 |
|  |  |  | KTDAALTR | 19.5 | 386 -393 |  |  |  |
|  |  |  | AINQVGQLK | 11.0 | 304 -312 |  |  |  |
|  |  |  | GQEGSYNIK | 10.4 | 236 -244 |  |  |  |
|  |  |  | VRDQFLDK | 12.5 | 76 -83 |  |  |  |
|  |  |  | ADPSQFTVSK | 7.33 | 340 -349 |  |  |  |
|  |  |  | DTAQAVANLSQK | 4.40 | 350 -361 |  |  |  |
|  |  |  | AFQSEMSLLEK | 5.74 | 163 -173 |  |  |  |
|  |  |  | AFQSEMSLLEK+ Oxidation (M) | 8.40 | 163 -173 |  |  |  |
|  |  |  | LIGIDASMSHVAK | 8.43 | 291 -303 |  |  |  |
|  |  |  | LIGIDASMSHVAK+ Oxidation (M) | 9.35 | 291 -303 |  |  |  |
|  |  |  | AFQSEMSLLEKER | 8.04 | 163 -175 |  |  |  |
|  |  |  | AFQSEMSLLEKER+ Oxidation (M) | 5.69 | 163 -175 |  |  |  |
|  |  |  | LIDELAILLPIEVER | 8.77 | 216 -230 |  |  |  |
|  |  |  | QFQLEAHVSGYTAVQSR | -41.64 | 84 -100 |  |  |  |
|  |  |  | IEESGQAANELYDQQDR | 2.56 | 199 -215 |  |  |  |
|  |  |  | EYQQMMGQLGVEAQSMQR | 9.61 | 368 -385 |  |  |  |
|  |  |  | EYQQMMGQLGVEAQSMQR+ Oxidation (M) | 0.84 | 368 -385 |  |  |  |
|  |  |  | EYQQMMGQLGVEAQSMQR+ 2 Oxidation (M) | 0.40 | 368 -385 |  |  |  |
|  |  |  | EYQQMMGQLGVEAQSMQR+ 3 Oxidation (M) | 0.28 | 368 -385 |  |  |  |
|  |  |  | EREGTEATINDLLEQIAINNR | -0.85 | 174 -194 |  |  |  |
|  |  |  | QQLMSEVNELVPNFFDVSTGR | -4.46 | 313 -333 |  |  |  |
|  |  |  | QQLMSEVNELVPNFFDVSTGR+ Oxidation (M) | 1.79 | 313 -333 |  |  |  |
| 36 | AIC96522 | Single-stranded DNA-binding protein | GSLAGVDGR | 5.65 | 68 -76 | 7/39 | 59 | 92 |
|  |  |  | LTRDPELR | -8.36 | 11 -18 |  |  |  |
|  |  |  | SYDNNEGKR | -16.29 | 81 -89 |  |  |  |
|  |  |  | KPAENVANFLK | -21.01 | 56 -66 |  |  |  |
|  |  |  | EADFINCVVWR | -24.94 | 45 -55 |  |  |  |
|  |  |  | VFITEVVAESVQFLEPR | -30.61 | 90 -106 |  |  |  |
|  |  |  | NSQNNQNQNFGGSQGNYGSGSGNTGSSGGNR | -44.68 | 107 -137 |  |  |  |
| 85 | AIC96549 | Inosine-5'-monophosphate dehydrogenase | EEGQFVR | -14.74 | 454 -460 | 17/43 | 35 | 136 |
|  |  |  | LVPEGIEGR | -19.89 | 411 -419 |  |  |  |
|  |  |  | VIEFPNSAK | -23.16 | 205 -213 |  |  |  |
|  |  |  | FIEDYSIK | -32.02 | 143 -150 |  |  |  |
|  |  |  | YFQENNQK | -32.22 | 403 -410 |  |  |  |
|  |  |  | VGIGPGSICTTR | -27.06 | 298 -309 |  |  |  |
|  |  |  | QVFDAEHLMGK | -39.10 | 107 -117 |  |  |  |
|  |  |  | QVFDAEHLMGK+ Oxidation (M) | -31.19 | 107 -117 |  |  |  |
|  |  |  | KHGVPIIADGGIK | -28.70 | 331 -343 |  |  |  |
|  |  |  | DRYFQENNQK | -29.63 | 401 -410 |  |  |  |
|  |  |  | AGMGYCGTATLTNLR | -34.62 | 439 -453 |  |  |  |
|  |  |  | AGMGYCGTATLTNLR+ Oxidation (M) | -34.11 | 439 -453 |  |  |  |
|  |  |  | SESGVITDPFFLTPDR | -35.84 | 91 -106 |  |  |  |
|  |  |  | FIEDYSIKIDDVMTK | -62.30 | 143 -157 |  |  |  |
|  |  |  | RSESGVITDPFFLTPDR | -38.03 | 90 -106 |  |  |  |
|  |  |  | AGADAIVIDTAHGHSQGVLNK | -42.52 | 241 -261 |  |  |  |
|  |  |  | YRISGVPIVNESQHLVGILTNR | -116.28 | 118 -139 |  |  |  |
| 100 | AIC94131 | Fumarate hydratase class II | IPFEVVR | -20.50 | 39 -45 | 10/42 | 26 | 72 |
|  |  |  | YWGAQTQR | -25.01 | 20 -27 |  |  |  |
|  |  |  | ENEFSDIVK | -31.78 | 174 -182 |  |  |  |
|  |  |  | DTIGEIQVPADK | -34.93 | 8 -19 |  |  |  |
|  |  |  | LLSDGLTNFHDK | -29.09 | 374 -385 |  |  |  |
|  |  |  | TERDTIGEIQVPADK | -25.37 | 5 -19 |  |  |  |
|  |  |  | CGIGEVIIPANEPGSSIMPGK+ Oxidation (M) | -31.47 | 303 -323 |  |  |  |
|  |  |  | HMLTTCSTMIEQSLDHVR+ 2 Oxidation (M) | -23.42 | 205 -222 |  |  |  |
|  |  |  | DTIGEIQVPADKYWGAQTQR | -17.88 | 8 -27 |  |  |  |
|  |  |  | ASENHFHGLTSHDELVYFHGALK | -24.61 | 259-281 |  |  |  |
| 123 | AIC96385 | Hypothetical protein, conserved | VEVNEQVVAK | -6.74 | 11 -20 | 6/36 | 80 | 79 |
|  |  |  | VSELGQEAVIK | -5.01 | 80 -90 |  |  |  |
|  |  |  | RVSELGQEAVIK | -4.20 | 79 -90 |  |  |  |
|  |  |  | FIAASVGETVSMEGIALLDDDGQSELKR+ Oxidation (M) | -24.24 | 52 -79 |  |  |  |
|  |  |  | LTSFTHSLEVEEADVTGLGDTVDGGGVFR | -22.56 | 21 -49 |  |  |  |
|  |  |  | HTDSTGSGYALTGFFTTYEEEGSVSEGVYTFSGEFR | -13.95 | 91 -126 |  |  |  |
| 126 | AIC95662 | Zinc D-Ala-D-Ala carboxypeptidase | AYQWTR | -11.47 | 2-7 | 7/33 | 49 | 132 |
|  |  |  | NGISGNDVR | -3.46 | 11-19 |  |  |  |
|  |  |  | VAGYAAATSSR | -5.16 | 25-35 |  |  |  |
|  |  |  | LGNIPLTINSGFR | 5.59 | 129-141 |  |  |  |
|  |  |  | FSGGAVSATQVQENVR | 4.6 | 101-116 |  |  |  |
|  |  |  | THITVDGQFGPATEGAVR | 7.29 | 36-53 |  |  |  |
|  |  |  | SHNSSVGGASNSQHLYGSAADVSSSTTPTR | -0.59 | 144-173 |  |  |  |
| 130 | AIC94787 | Polyribonucleotide nucleotidyltransferase | QANGAVLVR | 46.9 | 27 -35 | 16/42 | 24 | 120 |
|  |  |  | GILQMFVK+ Oxidation (M) | 33.9 | 294-301 |  |  |  |
|  |  |  | THGSGLFTR | 42.5 | 336-344 |  |  |  |
|  |  |  | EIGHGALGER | 36.0 | 399-408 |  |  |  |
|  |  |  | TFSVEVGQLAK | 16.0 | 16-26 |  |  |  |
|  |  |  | HEFTINWAGR | 12.2 | 6-15 |  |  |  |
|  |  |  | EVLEQALEQAK | 8.64 | 516-526 |  |  |  |
|  |  |  | TFDAELENALR | 14.3 | 237-247 |  |  |  |
|  |  |  | EGRPSEQAILTSR | 8.23 | 81-93 |  |  |  |
|  |  |  | ILDNMLTAIQEPR+ Oxidation (M) | 2.28 | 532-544 |  |  |  |
|  |  |  | REGRPSEQAILTSR | -0.65 | 80-93 |  |  |  |
|  |  |  | DLPFFPLTVNYEER | -0.67 | 53-66 |  |  |  |
|  |  |  | DQERHEFTINWAGR | 60.3 | 2-15 |  |  |  |
|  |  |  | GVDEIRPLDSQIHMLPR+ Oxidation (M) | -6.17 | 319-335 |  |  |  |
|  |  |  | ALEQVIPSEQEFPYTIR | -5.64 | 409-425 |  |  |  |
|  |  |  | AQVERYEEDETVEVSEVK | -2.64 | 276-293 |  |  |  |
| 131 | AIC93700 | Pyridoxal biosynthesis lyase pdxS | IGHIVEAR | 11.7 | 85 -92 | 9/40 | 30 | 65 |
|  |  |  | IGEGASMIR+ Oxidation (M) | 3.51 | 140 -148 |  |  |  |
|  |  |  | RIGEGASMIR+ Oxidation (M) | 2.67 | 139 -148 |  |  |  |
|  |  |  | DFTVPFVCGAR | -9.95 | 121 -131 |  |  |  |
|  |  |  | GEPGTGNIVEAVR | -2.18 | 151 -163 |  |  |  |
|  |  |  | GGVIMDVVNAEQAK+ Oxidation (M) | -10.97 | 20 -33 |  |  |  |
|  |  |  | TKGEPGTGNIVEAVR | -6.17 | 149 -163 |  |  |  |
|  |  |  | IAEEAGAVAVMALER+ Oxidation (M) | -10.54 | 34 -48 |  |  |  |
|  |  |  | AIVEATTHYKDYDLIAR | -15.23 | 251 -267 |  |  |  |
| 136 | AIC95662 | Zinc D-Ala-D-Ala carboxypeptidase | AYQWTR | -3.65 | 2 -7 | 9/86 | 73 | 74 |
|  |  |  | NGISGNDVR | 12.2 | 11 -19 |  |  |  |
|  |  |  | HFNWSEFHSR | -22.90 | 87 -96 |  |  |  |
|  |  |  | LGNIPLTINSGFR | -10.25 | 129 -141 |  |  |  |
|  |  |  | FSGGAVSATQVQENVR | -20.01 | 101 -116 |  |  |  |
|  |  |  | THITVDGQFGPATEGAVR | -16.24 | 36 -53 |  |  |  |
|  |  |  | TCGFSGIIIYQTFTHVDSR | -23.22 | 180 -198 |  |  |  |
|  |  |  | SHNSSVGGASNSQHLYGSAADVSSSTTPTR | -34.50 | 144 -173 |  |  |  |
|  |  |  | FQASYNLSADGVAGPQTHTALNNLEAADGSTR | -30.84 | 55 -86 |  |  |  |
| 148 | AIC93967 | Adenine phosphoribosyltransferase | DSIQPGQR | 31.1 | 106 -113 | 9/36 | 50 | 100 |
|  |  |  | AIMELTAYAK+ Oxidation (M) | 9.86 | 36 -45 |  |  |  |
|  |  |  | QQADVIVGPEAR | 12.1 | 47 -58 |  |  |  |
|  |  |  | KQQADVIVGPEAR | 5.13 | 46 -58 |  |  |  |
|  |  |  | DITTLMQNGPAYK+ Oxidation (M) | -2.76 | 22 -34 |  |  |  |
|  |  |  | EVIECDYGLEYGK | -9.08 | 86 -98 |  |  |  |
|  |  |  | FKDITTLMQNGPAYK+ Oxidation (M) | -12.71 | 20 -34 |  |  |  |
|  |  |  | GFVVGCPIATELELGFVPVR | -8.91 | 59 -78 |  |  |  |
|  |  |  | EVIECDYGLEYGKDCLTIHK | -15.11 | 86 -105 |  |  |  |

^a^ Spot number corresponding to spots in Figure 1

^b^ The AIC gene numbering is according to the NCBI taxonomy database for strain *B. lehensis* G1.

^c^ The annotation was primarily based on the genome annotation of *B. leheniss* G1
